# Glucagon-Like Peptide-1: A Focus on Neurodegenerative Diseases

**DOI:** 10.3389/fnins.2019.01112

**Published:** 2019-10-18

**Authors:** Maddalena Grieco, Alessandra Giorgi, Maria Cristina Gentile, Maria d’Erme, Susanna Morano, Bruno Maras, Tiziana Filardi

**Affiliations:** ^1^Department of Biochemical Sciences, Faculty of Pharmacy and Medicine, Sapienza University of Rome, Rome, Italy; ^2^Department of Experimental Medicine, Faculty of Medicine and Dentistry, Sapienza University of Rome, Rome, Italy

**Keywords:** glucagon-like peptide-1, GLP-1 receptor agonists, Parkinson’s disease, Alzheimer’s disease, neurodegenerative diseases, type 2 diabetes

## Abstract

Diabetes mellitus is one of the major risk factors for cognitive dysfunction. The pathogenesis of brain impairment caused by chronic hyperglycemia is complex and includes mitochondrial dysfunction, neuroinflammation, neurotransmitters’ alteration, and vascular disease, which lead to cognitive impairment, neurodegeneration, loss of synaptic plasticity, brain aging, and dementia. Glucagon-like peptide-1 (GLP-1), a gut released hormone, is attracting attention as a possible link between metabolic and brain impairment. Several studies have shown the influence of GPL-1 on neuronal functions such as thermogenesis, blood pressure control, neurogenesis, neurodegeneration, retinal repair, and energy homeostasis. Moreover, modulation of GLP-1 activity can influence amyloid β peptide aggregation in Alzheimer’s disease (AD) and dopamine (DA) levels in Parkinson’s disease (PD). GLP-1 receptor agonists (GLP-1RAs) showed beneficial actions on brain ischemia in animal models, such as the reduction of cerebral infarct area and the improvement of neurological deficit, acting mainly through inhibition of oxidative stress, inflammation, and apoptosis. They might also exert a beneficial effect on the cognitive impairment induced by diabetes or obesity improving learning and memory by modulating synaptic plasticity. Moreover, GLP-1RAs reduced hippocampal neurodegeneration. Besides this, there are growing evidences on neuroprotective effects of these agonists in animal models of neurodegenerative diseases, regardless of diabetes. In PD animal models, GPL-1RAs were able to protect motor activity and dopaminergic neurons whereas in AD models, they seemed to improve nearly all neuropathological features and cognitive functions. Although further clinical studies of GPL-1RAs in humans are needed, they seem to be a promising therapy for diabetes-associated cognitive decline.

## Introduction

The concern for neurodegeneration, a worldwide expanding set of diseases, stimulated the research on risk factors related to the lifestyle of the population, leading to interesting findings on the association between dysmetabolism and brain impairment. In this perspective, gut/brain axis and altered insulin release and response seem to be the main actors in establishing the pathological metabolic set up for the development of neurodegenerative diseases. Indeed, insulin works as pro-survival neurotrophic factor with its receptor widespread in cognitive areas as hippocampus and in dopaminergic system (Haas et al., 2016; Fiory et al., 2019).

In the last years particular attention has been devoted to unravel the role of glucagon-like peptide-1 (GLP-1), a gut released hormone that not only is one of the major components of the gut/brain axis, but it is also able to protect pancreatic beta-cells from apoptosis and to induce insulin secretion (Cabou and Burcelin, 2011).

Glucagon-like peptide-1 is an endogenous peptide hormone released by intestinal L-cells in response to meal. Gene expression generates pro-glucagon (PG), which is processed by prohormone convertases (PC1/3) to release the GLP-1 (1–37) peptide precursor (Sandoval and D’Alessio, 2015). Proteolytic cleavage and amidation of the precursor protein GLP-1(1–37) generate two GLP-1 active forms with the same biological activity namely GLP-1 (7–37) and the amidated GLP-1 (7–36). GLP-1 is degraded by a dipeptidyl-peptidase IV (DPP IV), a serine aminopeptidase expressed in the different organ, such as liver, pancreas, gut, and brain (Hopsu-Havu and Glenner, 1966; Smith et al., 2019). GLP-1 stimulates insulin secretion from the pancreatic beta-cells under hyperglycemic conditions and reduces glucagon secretion from the alfa-cells recovering insulin sensitivity and enhancing glycemic homeostasis (Meloni et al., 2013; Katsurada and Yada, 2016).

Glucagon-like peptide-1 signal transduction is mediated by GLP-1 receptor (GLP-1R), a G-protein coupled receptor, leading to cyclic adenosine monophosphate (cAMP) dependent activation of protein kinase A (PKA) and of cAMP-regulated guanine nucleotide exchange factor (Epac). The activation of Epac and PKA potentiates in a synergistic way the insulin release from the beta-pancreatic cells through phosporylation of the SNARE-associated protein Snapin and activation of L-type voltage gated calcium channels (Song et al., 2011).

It is noteworthy that GPL-1R may operate signal transduction even by activating the PI3K/AKT axis as observed in GLP-1 protection against apoptosis with the regulation of CREB and protein survival factors like Bcl-2 and Bcl-XL, through the action of β-arrestin-1 and the phosphorylation of ERK1/2. Furthermore, the activation of the PI3K/AKT axis can induce the inhibition of specific caspases and of NF-κB, with the resulting inhibition of the release of pro-inflammatory cytokines (Farilla et al., 2003; Athauda and Foltynie, 2016; Tramutola et al., 2017; Yang et al., 2018).

Plasticity of GLP-1 action at molecular level is mirrored also in different tissues like the cardiac compartment and the brain. GLP-1 plays a pivotal role preventing cardiovascular disorders, which makes GLP-1 and its analogs a great resource in the treatment of these diseases (Pozo et al., 2019). GLP-1 is also involved in the reduction of the oxidative stress, in the regulation of autophagy, and in the modulation of central nervous system (CNS) pathways with protective functions and elicitation of anti-inflammatory signaling (Li et al., 2009).

## Multifunctional Role of GLP-1

Glucagon-like peptide-1 is produced at neuronal level of the solitary tract within the brainstem. In addition, this peptide, released from the gut, activates the GLP-1R located on vagal sensory neurons that constitute the hepato-portal glucose sensor, communicating with brainstem neurons, extending its action to different brain regions. Several studies have shown the influence of GLP-1 on neuronal function such as thermogenesis, blood pressure control, neurogenesis, neurodegeneration, retinal repair, and energy homeostasis (Katsurada and Yada, 2016). Since GLP-1Rs are expressed in different brain regions, GLP-1 behaves as a neuropeptide, involved in different peculiar effects including the control of satiety, water intake, and stress reaction (van Dijk and Thiele, 1999; Meier et al., 2002). Kinzig et al. (2003) reported that GLP-1-stimulated brain GLP-1Rs are mediator of multiple stress responses. GLP-1 administration directly into the rat brain increases anxiety level, associated with a higher production of stress-activated hormones ACTH and corticosterone, demonstrating that GLP-1 is able to stimulate at the same time a response by both amygdala and by the paraventricular nucleus of the hypothalamus (Kinzig et al., 2003). The increase of GLP-1 in the circulation could reach the brain and regulate food intake (Ruttimann et al., 2009). Recent studies showed that GLP-1 in combination with dexamethasone (GLP-1/Dexa) can decrease food intake and lower body weight in obese mice without inducing mood or memory deficits (Decarie-Spain et al., 2019). In type 2 diabetes (T2D), alteration of insulin sensitivity and disturbances of neurogenesis are correlated with a reduction in GLP-1 levels in response to food, and its signaling activity (Nauck et al., 2011). Recently, several groups reported that GLP-1 contributes to the regulation of neurologic and cognitive functions (Muscogiuri et al., 2017). Indeed, GLP-1 is also involved in the control of the synaptic plasticity and in some forms of neuroprotection and thus has a regulative role in various signaling pathways associated with learning, memory, and other synaptic function (Gault and Holscher, 2008; Yildirim Simsir et al., 2018).

## Glucagon-Like Peptide-1 Receptor Agonists (GLP-1RAs) as Neuroprotective Agents in Diabetes-Associated Cognitive Impairment

Type 2 diabetes is a chronic disease with an increasing global prevalence. Besides the well-known micro- and macro-vascular complications, cognitive decline is thought to be an emerging consequence of diabetes (Koekkoek et al., 2015).

Over the last decade GLP-1RAs have emerged as effective glucose-lowering drugs. Exenatide was the first GLP-1RA approved for the treatment of T2D. It is a synthetic form of exendin-4, a natural GLP-1-like peptide extracted from the saliva of the lizard *Heloderma suspectum.* Exenatide shares 53% homology with native GLP-1. Exenatide has a half-life of 2.4 h, whereas GLP-1 has a half-life of 2 min. Lixisenatide is based on the structure of exendin-4 and has a half-life of 3 h. Liraglutide was the first GLP-1RA deriving from native GLP-1, sharing 97% homology and with a half-life of 13 h (Aroda, 2018). Semaglutide, a modified form of liraglutide has a half-life of ∼7 days due to a 5.6 times higher affinity with albumin than liraglutide (Gomez-Peralta and Abreu, 2019).

Several studies have investigated the neuroprotective actions of GLP-1RAs in animal models of diabetes. Many of them have focused on the effects of GLP-1RAs on cerebral ischemia/reperfusion injury. In diabetic rats with cerebral ischemia/reperfusion damage caused by middle cerebral artery occlusion, recombinant GLP-1 improved neurological deficit and reduced cerebral infarct area, mainly through the inhibition of oxidative stress and apoptosis (Fang et al., 2018). GLP-1RAs exert favorable effects, such as the reduction of cognitive impairment induced by diabetes or obesity.

Indeed, it has been observed that peripheral administration of lixisenatide for 40 days (50 nmol/kg bw, twice-daily) in high-fat fed mice with established obesity, insulin resistance, and impaired cognition resulted in marked improvement in recognition memory, which was associated with up-regulation of hippocampal expression of neurotrophic tyrosine kinase receptor type 2 and mammalian target of rapamycin (mTOR) genes involved in modulating synaptic plasticity and long-term potentiation. Lixisenatide treatment promoted also hippocampal progenitor cells proliferation and increased immature neurons in the hippocampal dentate gyrus (Lennox et al., 2014). Liraglutide showed effects against hippocampal neurodegeneration induced by streptozotocin (STZ), an animal model of diabetes and neurodegeneration associated with cognitive decline. In particular, liraglutide improved learning and memory, and reduced hippocampal neuronal death (Palleria et al., 2017). In addition, in a STZ-induced mouse model of diabetes, pre-treatment with liraglutide contrasted neuronal and synaptic damage in the hippocampal CA1 region (Kong et al., 2018).

Notably, neuroprotective activity of GLP-1RAs seem not to be entirely related to glycemia normalization. Indeed, there is growing evidence about neuroprotective effects of GLP-1RAs in animal models of neurodegenerative diseases, regardless of diabetes. Liraglutide reduced infarct size in the brain of diabetic and non-diabetic rats but decreased neurologic deficits only in non-diabetic rats, suggesting that the GLP-1 RAs effects on cognitive function are not associated with diabetes and glycemia normalization. Indeed, both liraglutide and metformin, a glucose lowering agent acting via AMP-activated protein kinase-dependent pathways, induced euglycaemia in diabetic rats, but only liraglutide treatment reduced ischemic brain damage (Filchenko et al., 2018).

## GLP-1RAs as Neuroprotective Agents in Neurodegenerative Diseases

Considering the beneficial effects of GLP-1RAs on neuropathological features it is conceivable a link between T2D and neurodegenerative disease such as Parkinson’s disease (PD) and Alzheimer’s Disease (AD). Neurodegenerative diseases have a considerable physical, psychological, social, and economical impact, both on affected people and on their careers, families, and society in general (World Health Organization, 2017).

### Parkinson’s Disease

Parkinson’s disease is a progressive nervous system disorder whose etiology remains still unclear, although genetic and environmental factors seem to be involved. PD’s clinical features include resting tremor, rigid muscles, slowed movement (bradykinesia), postural instability, and loss of purposeful movement (Kalia and Lang, 2015). Pathological features are characterized by neurons impairment of substantia nigra pars compacta with concomitant formation of intracellular Lewy bodies and loss of dopaminergic neurons. Lewy bodies are abnormal aggregates of α-synuclein protein, which is involved in dopamine (DA) metabolism and function. Dopaminergic neurons dysfunction and death by apoptosis or autophagy are also associated with mitochondrial activity alteration, oxidative stress, altered protein handling, and inflammatory condition (Olanow and Tatton, 1999). Rare dominant form of PD in familial and sporadic cases is associated to point mutations, duplications, and triplications in the α-synuclein gene (Lesage and Brice, 2009).

In different preclinical models of PD, GLP-1RAs showed neuroprotective effects, influencing motor activity, dopaminergic neurons, cortical activity, and energy utilization in the brain. Harkavyi et al. tested the efficacy of exendin-4 in rat models of PD treated with 6-hydroxydopamine (6-OHDA) and lipopolysaccaride (LPS) (Harkavyi et al., 2008). They observed that in striatal tissue DA concentrations were markedly higher in 6-OHDA/LPS + exendin-4 treated rats with respect to 6-OHDA/LPS + vehicle groups. This effect was associated with an increase in the tyrosine hydroxylase enzyme involved in the production of L-dopa, a DA precursor. In the same PD rat model exendin-4 was able to promote adult neurogenesis *in vitro* e *in vivo*, normalizing DA imbalance, showing an increase in tyrosine hydroxylase- and vesicular monoamine transporter 2-positive neurons in the substantia nigra (Bertilsson et al., 2008). Other authors observed that the administration of exendin-4, liraglutide, and lixisenatide in the same mouse model prevented both motor dysfunction and tyrosine hydroxylase levels reduction in the substantia nigra and basal ganglia. Furthermore, liraglutide and lixisenatide induced a marked increase in anti-apoptotic pathways compared to exendin-4 (Liu et al., 2015).

Recently, the long-term administration of liraglutide was found to rescue dopaminergic neuronal loss and motor impairment also in diabetic db/db mice, an established model of diabetes, with a mutation in the gene encoding the leptin receptor (Ma et al., 2019), suggesting that long-term injection of liraglutide might prevent motor function impairment and PD development also in patients with T2D. In rotenone-induced PD model, liraglutide together with sitagliptin, a DPP IV inhibitor, increased striatal DA and tyrosine hydroxylase protein levels, reduced neuroinflammation, and reversed neuronal loss (Badawi et al., 2017). Liraglutide was also able to attenuate dyskinesia, a serious complication of long-term therapy with L-dopa (Badawi et al., 2019).

In the MPTP mouse model of PD, semaglutide improved most of neuropathological features of PD, reversing motor impairment, inducing the increase of tyrosine hydroxylase levels, and attenuating neuroinflammation and apoptosis in the substantia nigra and striatum (Zhang et al., 2018). A reduction in α-synuclein aggregation occurred after this treatment, not observed with other GLP-1RAs (Zhang et al., 2019), highlighting semaglutide as an effective treatment for PD.

Preliminary clinical studies were performed with subcutaneous injections of exenatide in PD patients. Athauda et al. (2017) reported the results of the first randomized, double-blind, placebo-controlled trial in 62 patients affected by moderate PD. Patients were randomly assigned to receive subcutaneous injections of exenatide 2 mg once-weekly (n.32) or placebo (n.30) for 48 weeks. Exenatide had positive and sustained effects (12 weeks after exposure) on clinically assessed motor function. A *post hoc* analysis indicated that even non-motor symptoms, such as clinically evaluated mood and emotional well-being, improved in patients treated with exenatide although these beneficial effects did not last after interruption (Athauda et al., 2018). Patients treated with exenatide had significantly higher tyrosine phosphorylation of insulin receptor (IR) substrate 1 and higher expression of total Akt and phosphorylated mTOR than placebo-treated patients providing a possible insulin-based molecular mechanism explanation for the results observed in clinical trial (Athauda et al., 2019).

### Alzheimer’s Disease

Dementia is a chronic disease, which affects memory, other cognitive abilities and behavior. It is estimated that approximately 50 million people worldwide have dementia. Currently, it is the 7th leading cause of death and it is one of the major causes of disability worldwide. Pre-diabetic risk factors, obesity, and metabolic syndrome can promote cognitive dysfunction. AD is the most common form of dementia, contributing to 60–70% of cases. The main neuropathological features of AD are neurofibrillary tangles, formed by hyperphosphorylated tau proteins, which aggregate into oligomers, and the amyloid plaques, formed by aggregated β-amyloid peptides (Aβ) (Calsolaro and Edison, 2015).

Increasing evidence suggests a link between T2D and AD. In particular, these conditions might share defects in insulin signaling. Interestingly, in a mouse model of genetically induced AD-like neuropathology (3xTg-AD mice) peripheral glucose intolerance was observed. Treatment with pioglitazone, a glucose lowering drug, greatly improved cognitive impairment of these mice confirming the neurotrophic role of insulin (Masciopinto et al., 2012). In the same model, high-fat diet further potentiated glucose intolerance and enhanced neuropathological features of AD and memory deficits. Insulin adoption reversed the negative effect of high-fat diet, interrupting the vicious cycle between diabetes and AD (Vandal et al., 2014). Both studies highlighted the neurotrophic role of insulin in brain.

Conversely, hyperinsulinemia induced by peripheral administration of insulin increased tau phosphorylation by in C57BL/6 mice (Freude et al., 2005).

Aggregated β-amyloid peptide oligomers induced reduction of IRs activity due to the phosphorylation of IRS-1 at serine residues (IRS-1pSer), with a consequent loss of substrate affinity as observed in T2D. As reported by Bomfim et al. (2012), in the mouse hippocampal neurons Aβ oligomers are also thought to activate the TNF-α/JNK signaling, inducing insulin resistance (De Felice, 2013). The GLP-1RAs not only prevent JNK/IKK activation, but promote insulin activation by PI3K/AKT axis, with the subsequent activation of mTOR and the block of GSK-3β, an essential kinase also involved for the phosphorylation of tau protein (Moloney et al., 2010). Ma et al. (2015) reported that liraglutide administration prevented tau hyperphosphorylation associated with aging in diabetic db/db mouse.

The role of vascular dysfunction has recently emerged as significant contributor in the pathophysiology of AD. Blood–brain barrier and cerebral blood flow reduction might precede Aβ oligomers and tau deposition and it is associated to cognitive decline (Hachinski et al., 2019; Nation et al., 2019). In APP/PS1 transgenic mice liraglutide reduced the incidence of cerebral microanuerysms and leakage (Kelly et al., 2015).

Glucagon-like peptide-1 receptor agonists have shown neuroprotective effects in several preclinical studies in AD. Notably, they seem to improve nearly all neuropathological features in AD and cognitive functions as well. In 12-month-old female APP/PS1/tau AD mouse model, neurofibrillary tangles, amyloid plaques, and neuroinflammation in the hippocampi have been reduced by lixisenatide (Cai et al., 2018). In the rat model, lixisenatide also prevented synaptic damage induced by Aβ accumulation and strengthened spatial memory by affecting the PI3K-Akt-GSK3β (Cai et al., 2014). The GLP-1RA exenatide (20 μgr/kg/day, intraperitoneally for 2 weeks) reduced neuroinflammation by suppressing the TNF-α levels in rats. Furthermore, it improved memory and prevented the loss of hippocampal neurons (Solmaz et al., 2015).

Recently, it has been observed that 4-week-treatment with exendin-4 reversed memory impairment in APP/PS1 mice, downregulating the aberrant *N*-acetylglucosaminyltransferase III expression through the Akt/GSK-3β/β-catenin signaling pathway in neurons. *N*-Acetylglucosamine levels seem to be increased in the cerebrospinal fluid of most AD patients, and the levels of *N*-acetylglucosaminyltransferase III, a glycosyltransferase responsible for synthesizing a bisecting GlcNAc residue, were found to be highly expressed in the brains of AD patients as well (Wang et al., 2018).

Liraglutide (25 nmol/kg, intraperitoneally, for 2 months) improved spatial memory in 14-month-old APP/PS1 mouse model, compared to saline-treated mice. It also reduced inflammation and plaque load, while neuronal progenitor cell in the dentate gyrus increased. Long-term potentiation was significantly enhanced as well and synapse numbers increased in the hippocampus and cortex (McClean and Holscher, 2014). In another study, the same authors observed that liraglutide might also protect from progressive neurodegeneration that develops in AD: in 2-month old mice, liraglutide (once-daily intraperitoneally for 8 months) contrasted synaptic damage and improved memory. In addition, amyloid plaque load was reduced, inflammation was reduced in the cortex, and neurogenesis was enhanced in the dentate gyrus (McClean et al., 2015). On the contrary, other authors did not report beneficial effects of liraglutide on cerebral plaque load, in APP/PS1 transgenic mouse models of AD with two different clinical APP/PS1 mutations (Hansen et al., 2016). In the mouse model, memory deficit was improved by subcutaneous administration of liraglutide (25 nmol/day once daily for 8 week), decreasing the phosphorylation of tau (Qi et al., 2016).

Furthermore, in APP/PS-1 mice at different ages, chronic administration of liraglutide promoted neural progenitor cells proliferation. Both acute and chronic treatment increased the number of immature neurons in animals at all ages, and the differentiation into mature neurons was observed for most immature cells (Parthsarathy and Holscher, 2013).

Even in a mouse model of pathological aging, which shares neurobehavioral and neuropathological dysfunction with sporadic AD at an early phase, liraglutide increased the number of CA1 pyramidal neuron in hippocampus and improved memory (Hansen et al., 2015).

The effects of GLP-1RAs on synaptic protection might involve the modulation of the brain-derived neurotrophic factor (BDNF), a trophic factor which promotes neural progenitor cell differentiation and survival. Indeed, exenatide activates the transcription factor CREB with an increase of BDNF protein expression promoting the activation of neurotrophic pathway and inhibiting apoptosis in a mouse model of age-dependent cognitive dysfunction, potentiating long-term memory (Bomba et al., 2018). Even in a mouse model of AD (the 3xTg-AD undergoing high fat diet), exenatide reverted the impairment of BDNF signaling and neuroinflammation (Bomba et al., 2019).

In the last few years, even dual and triple receptor agonists have been developed, with remarkable results in animal models. Indeed, GLP-1/gastric inhibitory polypeptide (GIP) dual agonist DA5-CH strengthened working memory and long-term spatial memory in APP/PS1 transgenic AD mouse model (9-month-old). It also led to a reduction in hippocampal amyloid senile plaques and in phosphorylated tau protein. The deficits in hippocampal late-phase long-term potentiation were reversed and p-PI3K and p-AKT growth factor kinases were up regulated. The excessive activation of p-GSKβ was prevented in the hippocampus (Cai et al., 2018). Promising results have been observed with the dual GLP-1/GIP receptor agonist DA-JC4 as well, which decreased phosphorylated tau levels in the rat cerebral cortex and hippocampus, prevented spatial learning dysfunction, attenuated chronic inflammation response in the brain, reduced apoptosis, and reactivated insulin signaling pathways in STZ-induced AD rat model (Shi et al., 2017). Recently, a triple receptor agonist, activating GLP-1, GIP, and glucagon receptors, rescued memory dysfunction, showed anti-apoptotic effects, enhanced synaptophysin, protected from synaptic loss, reduced the total amount of Aβ, and reduced neuroinflammation (activated microglia and astrocytes) and oxidative stress in the cortex and hippocampus (Tai et al., 2018).

Despite the large amount of evidence about the neuroprotective effects of GLP-1RAs in animal models of AD, human studies are still scant. In a randomized, controlled, double-blind intervention study in AD patients, no effect on the deposition of Aβ was observed in patients treated for 6 months with liraglutide, compared to placebo (Egefjord et al., 2012). In a more recent 26-week, double-blind RCT, although glucose metabolism increased in multiple regions in patients with AD treated with liraglutide compared to placebo, the statistical power of the study was insufficient to reach a conclusion about Aβ load and cognition measures (Gejl et al., 2016).

## Conclusion

Overall, the results on the effects of GLP-1RAs in animal models of neurodegenerative diseases are encouraging. However, further clinical research is needed to clarify whether they might be potential agents for the treatment of PD and AD and other forms of cognitive impairment.

## Author Contributions

MD’E and SM conceptualized and critically revised the manuscript. TF, MG, and MCG performed the PubMed search and wrote the mini-review. BM and AG critically revised the article for intellectual content.

## Conflict of Interest

The authors declare that the research was conducted in the absence of any commercial or financial relationships that could be construed as a potential conflict of interest.
